# Screened moments and extrinsic in-gap states in samarium hexaboride

**DOI:** 10.1038/s41467-018-04007-z

**Published:** 2018-04-18

**Authors:** W. T. Fuhrman, J. R. Chamorro, P. A. Alekseev, J.-M. Mignot, T. Keller, J. A. Rodriguez-Rivera, Y. Qiu, P. Nikolić, T. M. McQueen, C. L. Broholm

**Affiliations:** 1 Institute for Quantum Matter and Department of Physics and Astronomy, The Johns Hopkins University, Baltimore, MD 21218 USA; 2 Department of Chemistry, The Johns Hopkins University, Baltimore, MD 21218 USA; 3 National Research Centre “Kurchatov Institute”, 123182 Moscow, Russia; 4 National Research Nuclear University “MEPhI”, 115409 Moscow, Russia; 5 Laboratoire Léon Brillouin, CEA-CNRS, CEA/Saclay, 91191 Gif sur Yvette, France; 6 Max Planck-Institut für Festkörperforschung, Heisenbergstrasse 1, D-70569 Stuttgart, Germany; 7 Max Planck Society at the Forschungsneutronenquelle Heinz Maier-Leibnitz (MLZ), D-85747 Garching, Germany; 8 Department of Materials Sciences, University of Maryland, College Park, MD 20742 USA; 9 NIST Center for Neutron Research, National Institute of Standards and Technology, Gaithersburg, MD 20899 USA; 10 School of Physics, Astronomy and Computational Sciences, George Mason University, Fairfax, VA 22030 USA; 11 Department of Materials Science and Engineering, The Johns Hopkins University, Baltimore, MD 21218 USA

## Abstract

Samarium hexaboride (SmB_6_) is a Kondo insulator, with a narrow gap due to hybridization between localized and conduction electrons. Despite being an insulator, many samples show metal-like properties. Rare-earth purification is exceedingly difficult, and nominally pure samples may contain 2% or more of impurities. Here to determine the effects of rare-earth doping on SmB_6_, we synthesized and probed a series of gadolinium-doped samples. We found a relationship between specific heat and impurity moment screening which scales systematically. Consistent with this finding, our neutron scattering experiments of a high purity sample of doubly isotopic ^154^Sm^11^B_6_ show no intrinsic excitations below the well-established 13 meV spin-exciton. The result of introducing impurities into a Kondo insulator is incompletely understood, but it is clear from our measurements that there is a systematic relationship between rare-earth impurities and metal-like properties in SmB_6_.

## Abstract (web summary)

The unconventional behaviour of samarium hexaboride has been difficult to explain in part because of differences between samples. Here the authors use gadolinium to exemplify that hard to avoid impurities introduce a low energy density of states that may explain earlier observations.

## Introduction

First prepared in the 1950s, SmB_6_ hosts exotic properties that seem to blur the lines between metal and insulator^1–7^. Early curiosities include its transition to an insulating state upon cooling, and that in contrast to other rare-earth hexaborides it has a strongly mixed-valence character with magnetic Sm^3+^ and non-magnetic Sm^2+^ in similar proportion^2, 8, 9^. A low-temperature resistance plateau was found below 6 K that further complicated the electronic description of SmB_6_^10^. Optical conductivity and specific heat both show a low temperature response more akin to a metal than a bulk insulator^4, 11^. Over time the low energy phenomena in SmB_6_ have become associated with in-gap states, with the gap loosely defined as 15–35 meV as seen via (e.g.) Raman spectroscopy (suppression of spectral weight below 35 meV), ARPES (4*f* binding energy of 15–20 meV), point contact spectroscopy (20 meV conductance peak), and STM measurements (20 meV gap in tunneling conductance DOS)^12–18^.

Recently, the advent of topological band theory led to the classification of SmB_6_ as a topological Kondo insulator, with experiments revealing metallic surface states as the origin of the low-temperature resistance plateau^19, 20^. Yet despite this insight from topology, experimental probes such as specific heat, optical conductivity, and quantum oscillations still indicate metal-like properties originating in the bulk of SmB_6_, even while advanced transport measurements reveal the distinctly insulating nature of the bulk^4, 5, 21, 22^. Exotic phenomena have been proposed to account for this seemingly electronically neutral yet magnetic low energy density of states, without a clear consensus^23–25^.

Research into the role of impurities in SmB_6_ spans decades^3, 20, 26–29^. Even in nominally pure starting materials, concentrations of other lanthanides must be expected as rare-earth elemental purification is limited by the similarity (chemical, electronegativity, and mass) of rare-earth elements. Previously reported inductive coupled plasma spectroscopy revealed nominally pure SmB_6_ with upwards of 2% (mostly lanthanide) doping on the Sm site^30^. Vacancies in SmB_6_, more frequently problematic for floating-zone grown crystals, shift valence towards magnetic *J* = 5/2 Sm^3+^ and may manifest similarly to magnetic impurities^31^. These impurities form defects in the coherent Kondo lattice, so called “Kondo holes.” Doping La as a Kondo hole into SmB_6_ produces an approximately T-independent Sommerfield coefficient and increased susceptibility^32, 33^. Theoretical studies propose non-trivial electronic states in the vicinity of non-magnetic Kondo holes, though little is known about their magnetic counterparts^34, 35^.

Even nominally pure samples show dramatic variation in physical properties. Reported Sommerfield constants range from below that of the non-magnetic analog LaB_6_ (2 mJ mol^−1^ K^−2^) to more than an order of magnitude larger (30 mJ mol^−1^ K^−2^)^28, 29^. The low-temperature rise in C/*T* has been claimed intrinsic, appearing with a steeper rise but with lower magnitude in an isotopically pure sample of ^154^Sm^11^B_6_^27^. However, ^154^Sm mass purification retains ^154^Gd, the only other stable isotope with mass 154, and isotopic samples are known to contain hundreds of parts per million magnetic impurities. Our focus on Gd doping thus allows our study to span to the highly dilute limit of a single impurity type commonly present in SmB_6_.

Gd is an interesting dopant because unusual effects involving Sm and Gd have been previously noted. While the Sm moment is dominated by its orbital contribution (which opposes its spin component), Gd has a much larger spin-only moment. Gd impurities in SmAl_2_ maintain ferromagnetic spin order with Sm, leading to a compensated ferromagnet with vanishing magnetization at Gd concentrations near 2%^36, 37^. Electron spin resonance studies of Gd-doped SmB_6_ show a strong negative g-shift for Gd and suggest that Gd impurities host an additional electron within a 4*f*^7^ 5*d*^1^ state for temperatures below 6 K^38, 39^. Previous X-ray absorption spectroscopy shows that Gd impurities are electron donors similar to La, the prototypical non-magnetic Kondo hole. Thus, 4*f*^7^ Gd^3+^ may be expected to act as a spinful Kondo hole^31, 34^.

Here we present experiments and analysis on nominally pure and Gd-doped SmB_6_ which reveal an impurity moment screening via magnetization that increases systematically with Gd content. The reduction in effective Gd moment scales the low-temperature specific heat, even accounting for that of our highly pure (0.04% Gd) ^154^Sm^11^B_6_ sample. Measuring the low energy regime of this doubly isotopic sample directly via magnetic neutron scattering reveals no indications of magnetism below the 13 meV spin-exciton. We infer that extrinsic defects such as rare-earth impurities and vacancies, present in varying quantities in all samples of SmB_6_, create a gapless continuum which dynamically screens impurity moments, despite even heavily doped samples remaining DC insulators.

## Results

### Magnetization

**Fig. 1:**
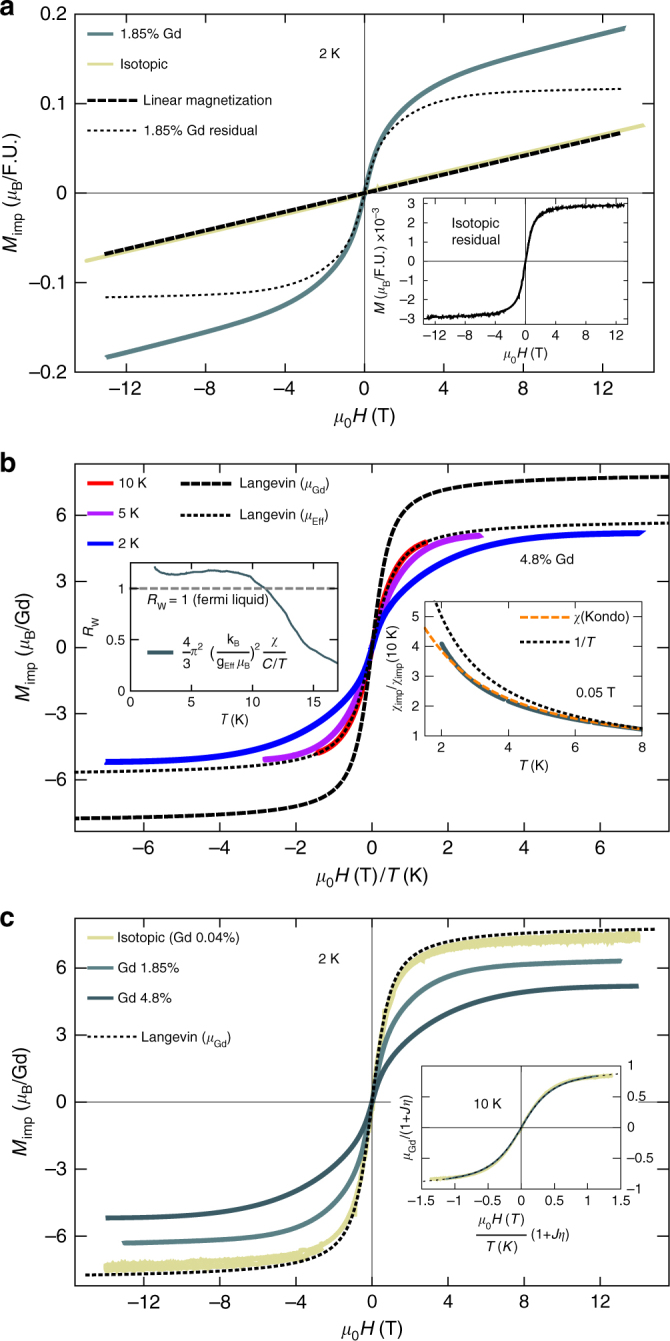
Magnetization of SmB_6_. **a** Raw magnetization of 1.85% Gd-doped sample and isotopic ^154^Sm^11^B_6_(colors). After removing a linear magnetization (dashed line) from both, the residual magnetization looks decidedly paramagnetic (dotted line in main figure shows 1.85%, inset shows isotopic on a smaller scale). We ascribe the linear contribution as intrinsic to SmB_6_ and the residual magnetization to impurities. Details of the fitting are included in Supplementary Note 1. **b** Residual magnetization of 4.8% Gd-doped sample at 2 K, 5 K, and 10 K, vs *μ*_0_*H*/*T*. Insets show the Wilson Ratio, *R*_w_, of this sample becoming approximately constant at low temperatures and the similarity of *χ*(*T*) to the Kondo impurity model rather than a Curie-like susceptibility (constant Van Vleck contribution has been removed). *g*_Eff_ in the *R*_w_ was determined by $g_{{\mathrm{Eff}}}\sqrt {J(J + 1)} = \mu _{{\mathrm{Eff}}}$. **c** Residual magnetization at 2 K of samples with varying doping levels. Inset shows that at 10 K a data collapse is achieved when scaling *M*_Imp_ and *H/T* with (1 + *Jη*) and 1/(1 + *Jη*), respectively

Given its large moment size, paramagnetic Gd is apparent in magnetization even at low concentrations and moderate field/temperature. After removal of the Van Vleck linear magnetization^5^, the residual magnetization of all our samples is strikingly paramagnetic, Fig. 1. We build our analysis on this component, which allows determination of both impurity concentration and effective paramagnetic moment (Supplementary Note 1).

**Fig. 2:**
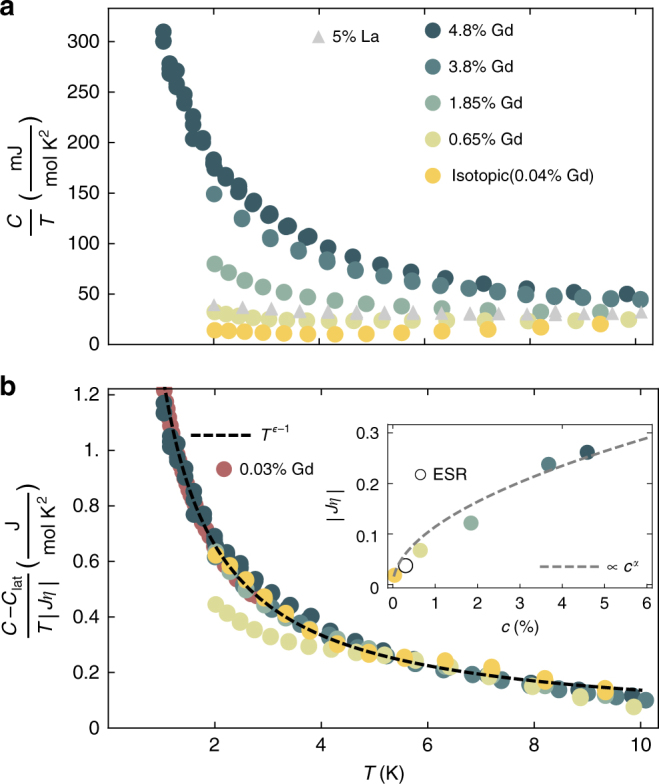
Heat Capacity of Gd and La-doped SmB_6_. **a** Raw heat capacity over temperature. The uptick is most dramatic in the heavily Gd-doped samples, while 5% La introduces a predominantly linear heat capacity at much lower magnitude per impurity than Gd doping. **b** Heat capacity of Gd-doped samples with lattice contribution (*βT*^3^) removed, then scaled by *Jη* as determined from magnetization data. The same value of *β* = 0.2 (mJ K^−4^ mol^−1^) is used for all samples, obtained from ref. ^41^. Previously published results for heat capacity of isotopic SmB_6_ at low temperatures are scaled and included in red^27^. The scaled *C/T* data is fit by *T*^ε−1^ where *ε* = 0.02. Inset shows *Jη* as a function of Gd concentration is proportional to *c*^*α*^ where *α* = 0.7(1)). Previously published ESR result (from g-factor shift) is included as an open circle^38^

Figure 1a shows the magnetization of the 1.85% Gd and isotopic (^154^Sm^11^B_6_) samples at 2 K. For Gd^3+^, S = 7/2 $(\mu _{{\mathrm{Gd}}} = 7.94\mu _{\mathrm{B}})$ and the classical limit for magnetization is applicable. The Langevin function describing paramagnetic magnetization is determined by the effective magnetic moment *μ*_Eff_ (field dependence and amplitude), and concentration *c* (amplitude only). Fitting the residual magnetization curves at 10 K yields effective moments and concentrations of (7.74*μ*_B_, 0.04%), (6.95*μ*_B_, 1.85%), and (5.84*μ*_B_, 4.8%) for isotopic and targeted 2% and 5% Gd-doped samples, respectively. Moment screening is unexpected for isolated Gd^3+^ given the half-filled 4*f*^7^ electron configuration, which carries no orbital moment and is particularly stable^40^. The applicability of the Langevin function for the rescaled residual magnetization of all samples at 10 K (Fig. 1c inset) is evidence that a reduced-moment model accurately describes the high-temperature (10 K) behavior for widely varying impurity concentrations. In addition to the reduced effective moment, we observe a departure from the paramagnetic Langevin behavior in the temperature-dependence of the magnetization, most evident in the 4.8% sample (Fig. 1b). The temperature range for this deviation coincides with an uptick in the linear portion of the specific heat, which is greatly enhanced with doping (Fig. 2a).

The enhanced linear specific heat and moment screening bear resemblance to the Kondo impurity effect seen in metals, and we choose this model to inform our intuition. In the *s*–*d* Kondo impurity model, magnetization and susceptibility are renormalized by *Jη*, a dimensionless parameter formed by the product of an exchange constant *J* and DOS *η* (Supplementary Note 4). To first order in *Jη*, the temperature independent correction to magnetization is $\mu _{{\mathrm{Eff}}} = \mu _{{\mathrm{Gd}}}(1 + J\eta )$ (Supplementary Eq. 20). The effective moments extracted at 10 K can thus be used to estimate *Jη* (inset of Fig. 2b). The Kondo susceptibility is given by $\chi = \chi _0(1 + J\eta (1 - J\eta \log\frac{{k_{\mathrm{B}}T}}{D})^{ - 1})$, where *D* is a half-width of the band providing the DOS at *E*_F_^42^. Using *Jη*=−0.26(1) from the magnetization fitting, the susceptibility of the 4.8% Gd-doped sample gives *D*=0.7(2) meV. This modifies the 1/*T* susceptibility, as seen in the deviation from the Langevin function at low *T* (Fig. 1(b)). In the Kondo model, this is a higher-order effect which indicates enhancement of Kondo screening at low temperatures. All samples showed enhanced screening at lower temperatures.

### Specific heat

After subtracting the lattice contribution ($\beta T^3,\beta = 0.2$ (mJ K^−4^ mol^−1^)), we scaled the specific heat by $1/|J\eta |$, Fig. 2b. For a constant *J*, this amounts to scaling by the moment screening DOS. The data converges to a single curve (the lightly doped but not isotopically purified 0.6% sample may not be dominated by Gd impurities). This scaling indicates the leading interactions are independent of doping, which is inconsistent with an origin in interactions between impurities. In our 5% targeted La-doped sample, we observed a linear heat capacity ≈ 40 mJ/mol-K^2^, similar to previous studies of electron-doping via carbon impurities. This is dramatically less than the low temperature heat capacity observed for our 5% Gd-doped sample, which rises above 300 mJ/mol-K^2^^41^. This indicates the involvement of impurity magnetism in the low energy DOS of Gd-doped samples.

The scaled specific heat has power-law dependence, *T*^*ε*^$({\mathrm{C}}/{\mathrm{T}} \propto T^{\varepsilon - 1})$, where *ε*=.02(1), Fig. 2b. Previously published specific heat data can be scaled to follow the same power law fit to 0.4 K, below which the power law fit diverges above the measured specific heat^27^. To first order in *ε*, $T^\varepsilon = 1 + \varepsilon \log(T)$, so the data is qualitatively consistent with the Kondo impurity model $C_{{\mathrm{Kondo}}} \propto 1 + 4J\eta \log(k_{\mathrm{B}}T/d)$^42^. However, *s*–*d* Kondo models have corrections to specific heat with amplitude $\propto (J\eta )^4$ whereas Fig. 2b shows the specific heat scales as $C \propto J\eta$. Nonetheless, the *Jη* scaling collapse of specific heat ties the low temperature DOS to the reduced impurity moment. Even the low temperature specific heat of our highest purity sample (0.04% Gd) is accounted for by this impurity component. The inset of Fig. 2b shows *Jη* scales approximately with *c*^*α*^, where $\alpha = 0.7(1)$. For comparison, theory predicts impurities produce an impurity band with DOS $\eta \propto \sqrt c$^34, 35^.

**Fig. 3:**
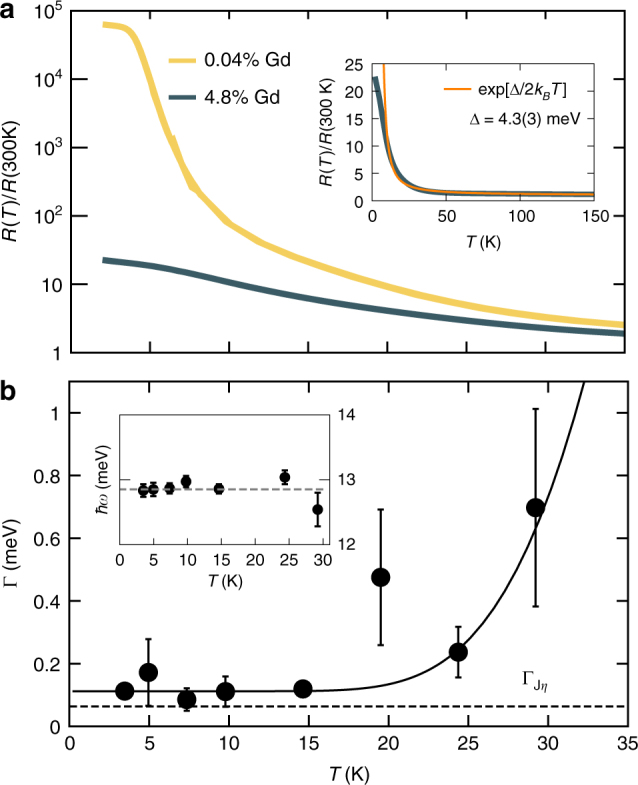
Normalized resistance and exciton lifetime. **a** Resistance of 4.8% Gd-doped (5.5 × 1 × 1 mm^3^ rod) and isotopically purified ^154^Sm^11^B_6_ (2 × 2 × 1 mm^3^ chip). In the doped sample, R(T) increases monotonically with decreasing T. Inset shows R(T) for the 4.8% Gd sample on a linear scale, which above 10 K fits to an exponentially activated form with a gap of 4.3(4) meV (orange line). **b** Spin-exciton lifetime and mode energy (inset) vs *T*. Solid line shows $\Gamma _0 + A\exp( - \Delta /k_{\mathrm{B}}T)$), with non-thermal lifetime $\Gamma _0 = 0.11$ meV and temperature dependence from exponential activation to the mode energy Δ = 12.8 meV). Dashed line is the lifetime estimated from coupling the collective mode to a DOS at *EF* ($\Gamma _{J\eta } = 0.080$ meV). Error bars described in Methods

For Fermi liquids, The Wilson ratio (*R*_w_) (inset of Fig. 1b) of the spin susceptibility and the specific heat over temperature tends to a constant in the low-T limit (Supplementary Note 3)^43^. For the 4.8% impurity concentration in SmB_6_, we find *R*_w_ approaches a constant near unity (Fig. 1b). This Fermi-liquid like *R*_w_ is another metal-like property of SmB_6_, unexpected because all samples show exponentially activated bulk DC resistivity indicating charge is ultimately localized (Fig. 3a).

### Neutron scattering

The long but finite lifetime of the spin-exciton in SmB_6_ (Fig. 3b) may also be connected to impurity induced low energy states. The spin-exciton arises from the coherent Kondo lattice, with a theoretically infinite lifetime imbued by interactions, and is sensitive to impurities and defects^44–46^. We utilized the same doubly isotopic sample as above on the hybrid triple-axis/spin-echo spectrometer TRISP to obtain the lifetime of the spin-exciton mode at the R-point (1/2 1/2 1/2)^47, 48^. The temperature dependence of the relaxation rate was modeled as the sum of an intrinsic width and exponential activation across a gap: $\Gamma _0 + A\exp( - \Delta /k_{\mathrm{B}}T)$. Fixing the gap energy to the exciton mode energy of 12.8 meV gives an intrinsic width of 0.11(1) meV and bandwidth *A*=0.7(2) eV; fitting both gap and intrinsic lifetime gives the same intrinsic width and uncertainty, though with larger error bars on Δ and *A* (19(5) meV and 4(17) eV, respectively). In an analogy to spin-resonance modes in cuprate superconductors, coupling of the exciton to a Fermionic DOS at *E*_F_ leads to a relaxation rate $\Gamma _0 = 4\pi (J\eta )^2\Omega$, where *J* is an exchange interaction ($J \propto t^2/U$ for the Hubbard model), *η* is the DOS, and Ω is the mode energy^49^. Given the relatively flat 4*f* bands in SmB_6_, coupling of the exciton to *η* may be similar to the Kondo coupling *J*, and so *Jη* provides an estimate of *Jη*. Substituting $J\eta = J\eta _{{\mathrm{isotopic}}} = - 0.0227$ and the mode energy Ω=12.8 meV yields Γ_*Jη*_ = 0.080 meV, which is similar to the measured value of 0.11(1) meV. The nearly temperature independent lifetime from 3.5 to 15 K suggests no coupling to additional energy scales below the 13 meV spin-exciton. A dramatic effect of doping on the spin-resonance mode and thermopower in the Tm-doped Kondo insulator YbB_12_ has been reported, and such studies are also warranted for Gd-doped SmB_6_^50^.

**Fig. 4:**
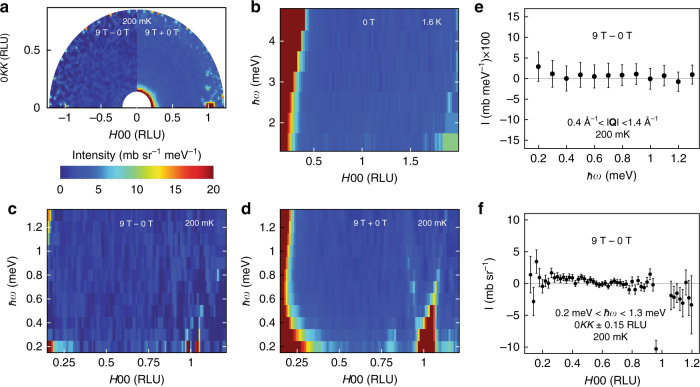
Low energy neutron scattering of SmB_6_ taken at 1.6 K and 200 mK. **a** Energy integrated from 0.2 to 1.3 meV. Left shows 9 T data with the 0 T data subtracted (9 T−0 T). Right shows the data sets averaged (9 T + 0 T). No substantial scattering is apparent in either, aside from the Bragg tail at 100 and low $|{\bf Q}|$ background scattering. **b** 0 T data from 1.5 meV to 5 meV. Only residual Bragg/phonon scattering is present. **c** 9 T−0 T data. There is no discernible field dependence of the low energy scattering. **d** Average of 9 T and 0 T data. The only substantial signal is residual Bragg scattering and weak phonon scattering at (100), along with a background powder ring at 0.8 RLU (seen as a very weak ring in the right side of (**a**). Again, no magnetic scattering is evident. **e** 9 T−0 T Scattering integrated from $0.4 \AA^{ - 1} < |{\bf Q}| < 1.4 \AA^{ - 1}$. This includes all scattering in the HKK plane between the low angle limitation of the instrument and the first Bragg peak, and shows no evidence of magnetic scattering. **f** 9 T−0 T scattering integrated from 0.2 meV to 1.3 meV. No field-dependent scattering is evident. The only substantial deviation from zero occurs at the 100 Bragg peak, attributed to counting statistics as seen in **c**. Error bars described in Methods

Consistent with the temperature dependence of the exciton described above, we observe no indications of magnetism in low energy neutron scattering of the doubly isotopic sample at both zero and high field (9 T). Figure 4 shows the scattering in the HKK plane of the doubly isotopic sample used to determine the exciton lifetime and in physical property measurements. Aside from residual nuclear Bragg scattering due to the spectrometer resolution, no **Q**, $\hbar \omega$, or field dependence of the scattering cross section is observed. The overall scattering cross section can be used to bound the total low energy fluctuating moment (Supplementary Note 2). The total moment squared is $(\mu _{{\mathrm{Eff}}}/\mu _{\mathrm{B}})^2 = {\int} {\int} Tr(S^{\alpha \beta }d^3{\bf Q}\hbar d\omega )/{\int} \mathrm{d}^3{\bf Q}$. Integrating over the first Brillouin zone for energies below the exciton (0.1–12.8 meV) we obtain a conservative upper bound of $< m_{{\mathrm{Eff}}}^2 > \le 0.05(2) \mu _{\mathrm{B}}^2$ on any fluctuating moment in the sub-exciton energy range. For comparison, the total fluctuating moment associated with the exciton is $< m_{{\mathrm{exciton}}}^2 > = 0.29(6) \mu _{\mathrm{B}}^2$ and 0.04% Gd would introduce a moment squared of $0.025 \mu _{\mathrm{B}}^2$^51^. We note that other sources of background include incoherent phonon scattering and the sample environment itself, and that the total moment of the exciton is contained within a comparatively narrow energy and momentum range.

## Discussion

The observed magnetization is evidence of doped magnetic moments. In zero field, isolated moments cannot store heat, yet we observe a non-thermally-activated contribution to specific heat coinciding with reduction in moment and dependent on doping concentration. The absence of an apparent energy scale down to 1 K and concurrent impurity moment screening leads us to infer that Gd moments are coupled to additional degrees of freedom with a gapless (Δ<1 K) spectrum. Although SmB_6_ is non-metallic, the Kondo impurity model provides a qualitative aid in the description of our magnetization and specific heat data. The intermediate valence intrinsic to SmB_6_ may facilitate electronic and magnetic fluctuations of magnetic Sm^3+^ and non-magnetic Sm^2+^^52^ adjacent and exchange-coupled to Gd^3+^ impurities, resulting in a dynamic screening of localized Gd moments which mimics the well-known Kondo impurity effect. Whether a Kondo impurity-like effect or another dynamic exchange mechanism, the net result is an insulator with screened impurity moments and substantial low energy DOS as evidenced by specific heat and magnetization.

The moment-screening effect we observe may manifest in other measurements. In strong magnetic fields, valence Landau levels periodically enhance the low energy DOS (LEDOS) which acts to screen impurity moments, giving rise to dHvA but not SdH oscillations via periodic impurity moment screening with frequency set by the pseudo Fermi surface of the sharply dispersing *d* bands^5, 51, 53^. The dramatic enhancement of quantum oscillation amplitude for floating zone grown samples compared to flux samples may arise from Sm vacancies, frequently introduced in floating zone growth. Samarium vacancies in SmB_6_ shift valence towards Sm^3+^ and may act as magnetic impurities^31^. Quantum oscillations in other Kondo insulators could reveal the pseudo Fermi surface, which itself is defined by band inversion in Kondo insulators, the locations of which fully specify topological invariants in inversion-symmetric Kondo insulators^54^. The substantial DOS indicated by specific heat and the approximately constant *R*_w_ is also consistent with the optical conductivity which, although large, is orders of magnitude smaller than for heavy Fermion metals.

Our neutron scattering results are important for constraining theories of the intrinsic low energy magnetism in SmB_6_, as well as for interpretation of other experimental results. In addition to systematic variation of physical properties with doping, we find no apparent magnetic excitations below the long-lived 13 meV spin-exciton in high-purity SmB_6_. Magnetic neutron scattering is well-suited for probing magnetic excitations such as those proposed for SmB_6_ (e.g., spinons, excitons, etc.), and our experiments place a tight upper limit on any excitations with a 4*f* electron form factor. Given the nearly complete Sm^3+^ moment contained within the spin-exciton^51^ and the continued trend to lower heat capacity with improved sample quality, it is reasonable to assign most if not all of the low energy DOS typically observed in SmB_6_ to extrinsic origins. The variety of metal-like properties induced by impurities that we have documented even in nominally pure samples is an important part of the phenomenology of this cornerstone Kondo insulator.

## Methods

### Crystal growth

Targeted 1%, 2%, and 5% samples were grown by the aluminum flux growth method in which elemental Sm (Ames Laboratory), Gd (Ames Laboratory), La (Ames Laboratory), B (Alfa Aesar 99.99%), and Al (NOAH Technologies 99.99%) were added to an alumina crucible in a 1−*x*:*x*:6:600 molar ratio, with *x* set to the desired (Gd, La) content. Al was premelted in the crucible prior to combining target elements and adding additional Al, which was then heated to 1350 °C at a ramp rate of 100° per h. The temperature was held for 12 h and then allowed to cool to 800°C at a rate of 4.5°C/hr, followed by cooling to room temperature at 100° per h. The aluminum flux was etched off with a caustic NaOH solution, and the crystals were isolated via vacuum filtration. Resultant crystals were phase-pure(Supplementary Note 1), and rod shaped with typical dimensions up to 11 × 1 × 1 mm^3^. The isotopic ^154^Sm^11^B_6_ sample was grown by Yu Paderno at IPMS (Kiev) by the floating zone method and is the same sample used in other neutron spectroscopy studies^51, 55^.

### Measurements

In-house powder x-ray diffraction measurements were performed to determine phase purity using Cu *Kα* radiation on a Bruker D8 Focus diffractometer with a LynxEye detector. Physical properties characterization was performed on a Quantum Design Physical Properties Measurement System (PPMS).

Both neutron scattering studies were performed on the same doubly isotopic ^154^Sm^11^B_6_ sample (labeled “isotopic” in PPMS measurements) described above. The lifetime of the spin-exciton was determined through inelastic neutron scattering performed on the TRISP hybrid triple-axis/neutron spin-echo spectrometer, utilizing a PG (002) monochromator and velocity selector with spin-echo coils in scanning DC operation for an energy resolution of 10 μeV, which was subtracted in quadrature from the measured width to obtain the physical width Γ (Supplementary Note 2). Details of the instrument and technique are complex and are summarized elsewhere^47, 48^. The low-energy scattering in high-field and zero field was performed on the high-flux cold neutron spectrometer MACS at the NIST NCNR^56^. We utilized cooled Be and BeO filters before and after the sample, with fixed final energies of 2.4 and 3.7 meV. Error bars indicate range for one standard deviation σ above and below the mean experimental result.

Certain commercial equipment, instruments, and materials are identified in this paper to foster understanding. Such identification does not imply recommendation or endorsement by the National Institute of Standards and Technology, nor does it imply that the materials or equipment identified are necessarily the best available for the purpose.

### Data availability

The materials and data which support the findings of this study are available from the authors on reasonable request.

## Electronic supplementary material

Supplementary Information
