# Supplementary material for: Screened moments and extrinsic in-gap states in samarium hexaboride
Source: Nat Commun. 2018 Apr 18;9:1539. doi: 10.1038/s41467-018-04007-z (PMC5906653; doi:10.1038/s41467-018-04007-z)
Supplement: Supplementary file 1 — Supplementary Information [file 41467_2018_4007_MOESM1_ESM.pdf]

Supplementary Note 1: Additional Data

Refinement of X-ray diffraction data

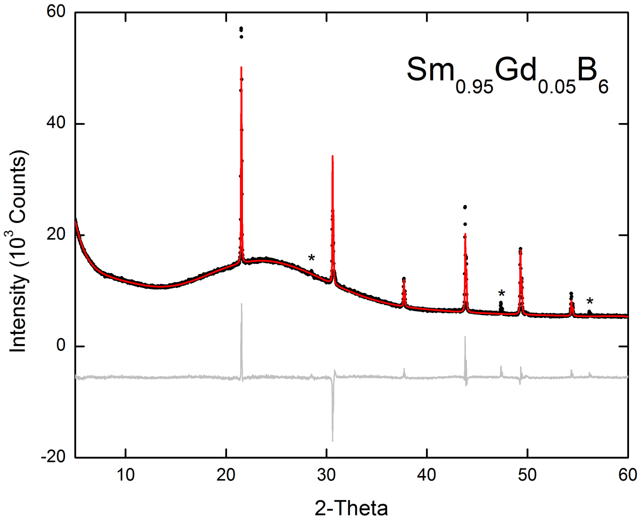

Supplementary Figure 1. Refinement of a 4 hour powder x-ray diffraction pattern collected at room temperature with Cu K-α radiation. Asterisks are at Bragg peaks of Si standard. Sample mass was approximately 5 mg.

Rietveld refinements were performed on x-ray diffraction data from grown crystals after grinding. No peaks corresponding to other Sm-B phases nor Sm rich or deficient phases were detected, and this places a 0.05% limit on second phase content. Due to the very small amounts of Gd used to dope the SmB_6 and the robustness of the hexaboride structure to varying rare-earth elements, no change in the lattice parameters is observed across flux grown samples to an accuracy of 1% $\Delta a/a$. . Results of the fit, which accounts for the large background arising from the sample platform, are in Supplementary Table 1.

Temperature	300 K
Space group	$Pm\bar{3}m$
a (Å)	4.1329(1)
Vol (Å^3)	70.59(5)
Sm	1 (1/2,1/2,1/2)
U_{iso} (10^2 Å^2)	10.5(2)
B	6 (0, 0, x); x = 0.50008(2)
U_{iso} (10^2 Å^{-2})	12.76(7)
R_p (%)	1.62
R_{wp} (%)	3.70
χ^2	3.71

Supplementary Table 1. Results of Rietveld refinement of 5% target Gd doped SmB_6.

Magnetization

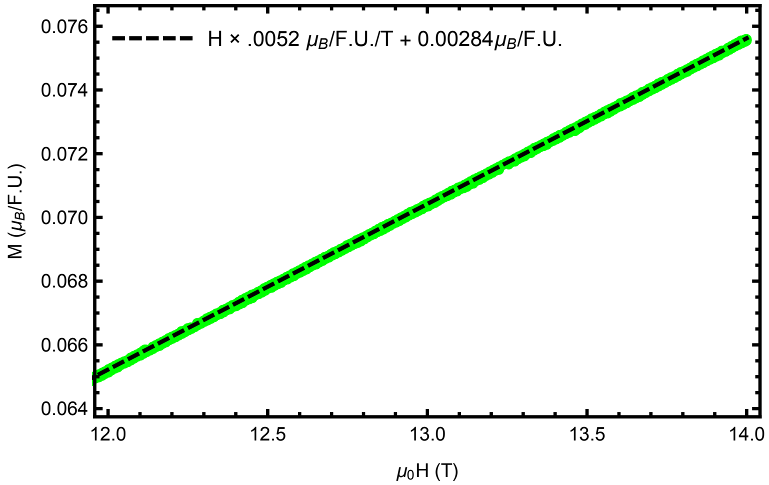

Supplementary Figure 2. High field magnetization of $^{154}Sm^{11}B_6$ with a fit to an offset linear function. We attribute the offset to the saturated moments of Gd impurities.

To determine the intrinsic contribution from SmB_6 to magnetization, we first fit the low-temperature, high-field regime of the most pure sample at our disposal, the isotopic $^{154}Sm^{11}B_6$ sample, to a simple linear form $M = \chi H + M_{\mathrm{Imp}}$ (Fig. 2). We ascribe the intercept $M_{\mathrm{Imp}} = 0.00284$ μ_B/F.U. to a saturated impurity magnetization and $\chi = 0.0052$ μ_B/F.U./T to the linear magnetization response previously reported up to 60 T in SmB_6.[1] Such a prescription is most robust for low-impurity levels with high effective magnetic moments where potential interactions are weak, and becomes less reliable if the full moment is not saturated (e.g. higher temperatures, larger interaction scales, smaller moments, etc). We proceed to fit a Langevin function with free parameters of effective moment and concentration plus a linear contribution. The magnetization per formula unit is fit by

$$M(h,\beta) = \chi_{bulk} h + c_{Imp}\mu_{Eff}(\coth(\mu_{Eff}\beta h) - \frac{1}{\mu_{Eff}\beta h}), \quad (1)$$

where χ_{bulk} is the constant of bulk susceptibility, $\beta = 1/k_B T$, and $\mu_{Eff} = \mu_{Gd}(1-|J\eta|)$. The linear magnetization is weakly temperature dependent below 10 K and can be associated with a van Vleck paramagnetic response of the Sm^{2+} state. The correction in the bulk susceptibility due to a change in Sm occupancy with doping is small on the scale of the impurity magnetization, which greatly increases with c_{Imp} due to the large moment size of Gd. Fig.3 shows the effectiveness of this fitting routine at 10 K for representative samples.

A reduced effective moment model for spin-1/2 magnetization is:

$$m = (1-|J\eta|)\tanh[(1-|J\eta|)\beta h/2] \quad (2)$$

For small $J\eta$ this is

$$m = (1-|J\eta|)(\tanh(\frac{\beta h}{2}) - \frac{|J\eta|\beta h}{2\cosh^2(\frac{\beta h}{2})}), \quad (3)$$

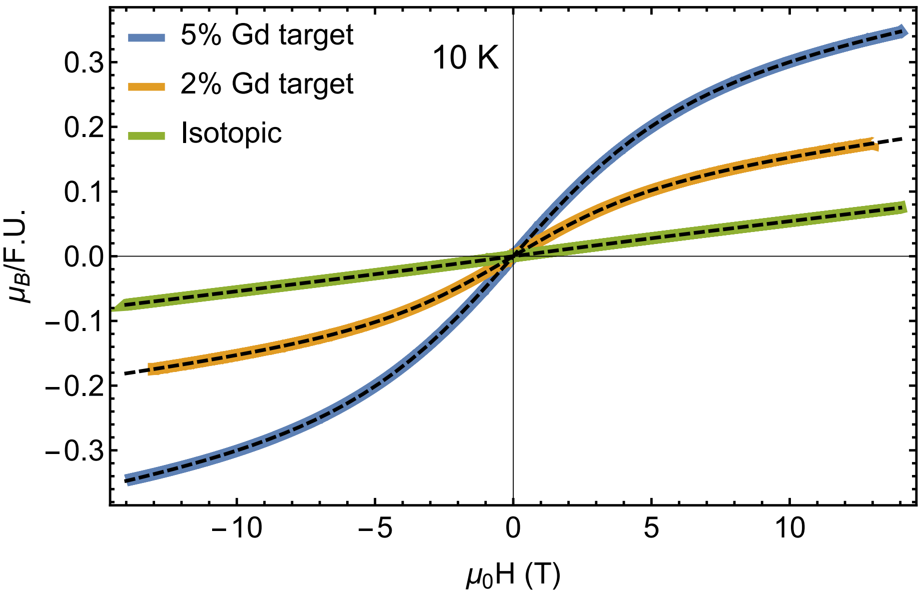

Supplementary Figure 3. Raw magnetization of the samples used in figure 1 in the main text. Each fit is the sum of a linear term and a reduced effective moment Langevin function, eq. 1.

which matches the Kondo s-d model result to first order in $J\eta$ (eq. 20). While we observe a clear reduction in moment, the sign of the Kondo coupling allows for enhancement or reduction in effective moment. For fitting higher S as appropriate for Gd impurities, we utilize an effective moment Langevin function (eq. 1).

Supplementary Note 2: Neutron Scattering

Conversion to absolute units

Our low-energy neutron scattering revealed no obvious magnetic scattering. By normalizing the measured scattering rate these data establish an upper limit on the total fluctuating moment in the dynamic range of the experiment. Various standard cross sections can be used to convert to absolute units, including incoherent elastic scattering from the sample, coherent acoustic phonon scattering, Bragg scattering from the sample, and incoherent elastic scattering from a known quantity of vanadium. We utilize the latter two cross sections. For vanadium scattering, we have per f.u. scattering of

$$\frac{d^2\sigma.}{d\Omega dE} = \frac{\sigma_v}{4\pi}\delta(\hbar\omega), \tag{4}$$

where σ_v is the incoherent scattering cross section for vanadium (5.1 barn). The corresponding monitor-normalized incoherent elastic vanadium scattering is given by

$$\widetilde{I}_v(\hbar\omega) = N_{van}\mathscr{C}\frac{\sigma_v}{4\pi}\frac{1}{\sqrt{2\pi}\sigma_E}\exp\big(\frac{\hbar\omega}{\sigma_E}\big)^2, \tag{5}$$

where N_{van} is the number of vanadium unit cells (scattering centers), $N\mathscr{C}$ is a normalization factor accounting for instrumental efficiencies, and σ_E is the energy resolution. Knowing the vanadium and

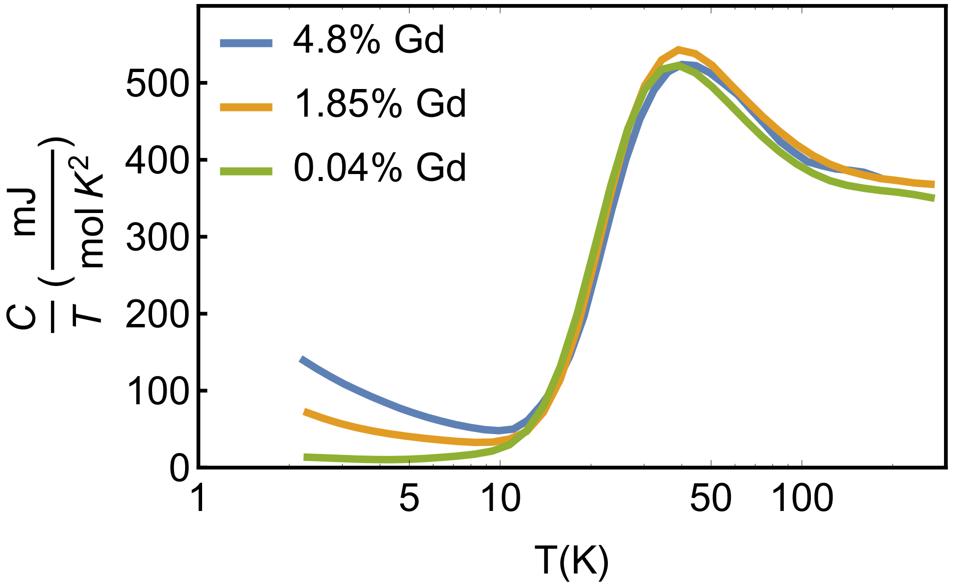

Supplementary Figure 4. Specific heat through the full temperature range. The broad maximum at 40-50 K persists through the range of doping, which indicates the resilience of the Kondo lattice insulator to doping. The slightly enhanced linear offset at higher temperatures in the doped samples may originate from the additional impurity band. The Kondo lattice increase in resistance is still apparent even in the 5% doped sample.

sample masses and intensity of vanadium scattering allows us to remove the unknown scattering efficiency factor to report the cross section in absolute units,

$$\frac{d^2\sigma_{SmB_6}}{d\Omega dE} = \frac{\widetilde{I}_{SmB_6}}{\int \widetilde{I}_{van} dE} \frac{N_v \sigma_v}{N_{SmB_6} 4\pi} \tag{6}$$

This normalization can be checked against the incoherent scattering coming from the sample itself by simply substituting the sum of the sample incoherent scattering cross section fort that of vanadium and comparing to incoherent elastic scattering (away from a Bragg peak). Doing so gives $N\mathscr{C}_{SmB_6}/N\mathscr{C}_v = 1.28$. The additional contribution to incoherent scattering seen from the sample may arise from the sample environment. The much stronger incoherent cross section from vanadium mitigates this effect since vanadium contributes a much larger portion of the total scattering.

Elastic Bragg scattering can also be used to estimate the accuracy of the normalization. In our experimental configuration, we were only able to access the 100 Bragg peak. The cross section for nuclear Bragg scattering is

$$\frac{d^2\sigma}{d\Omega dE} = N V_{BZ} \left|F_{100}\right|^2 \delta(E, \mathbf{Q} - \tau), \tag{7}$$

where V_{BZ} is the volume of the Brillouin Zone, $|F_{100}|$ is the structure factor for the 100 peak, and the delta function is at the 100 elastic Bragg position. Integrating over the total intensity scattered into this peak eschews complications from the resolution of the spectrometer. Using the normalization determined

from vanadium scattering, we recover 59% of the total intensity expected for the 100 Bragg peak. This discrepancy may originate in the unknown absorption of the sample (from, e.g. isotopes other than ^{154}Sm), or errors associated with the normalization process. These values are in agreement with the typical error associated with normalization by incoherent scattering of $\approx 30\%$.

Determination of mode lifetime by triple-axis spin-echo neutron scattering

The neutron spin-echo technique relies on the the precession of the neutron's magnetic moment in applied magnetic field, with the the phase of the precessing neutron acting as a high resolution measurement of the neutron velocity. Neutrons are subjected to magnetic field before and after scattering, and analysis of the field's affect on neutron polarization allows determination of small changes in the neutron velocity from which mode energy and width can be fitted. In the DC coil operation utilized in this experiment, neutrons traverse two coils of width L, before and after the sample. The precession frequency is $\omega_L = 2\pi\gamma B$, and the precession phase of a neutron traveling at velocity v for time $T = L/v$ through the field B is $\phi = 2\pi\gamma BL/v$, where γ is the gyromagnetic ratio of the neutron, 2.916 kHZ/G. The polarity of the pre and post scattering fields relative to the spin is opposite, and so the total precession phase collected is

$$\Delta\phi = 2\pi\gamma BL(\frac{1}{v_1} - \frac{1}{v_2}). \tag{8}$$

The designation of "spin echo" refers to elastic scattering where the phase shift vanishes and neutrons emerge with polarization matching their initial condition. Energy transferred in the scattering process at the sample results in a change in the kinetic energy of the neutron and results in a phase shift of the precession from the second coil relative to the first,

$$\Delta\phi = \frac{2\pi\hbar BL}{m_n v^3}\omega = \tau_{NSE}\,\omega. \tag{9}$$

τ_{NSE} is the "spin-echo time," and is a measure of resolution of the spectrometer. For a set coil length, adjusting B and v modulate the instrumental resolution. A mode with a finite width will result in a distribution of energy transfers centered about the peak mode energy. The average polarization is given by

$$P(\tau_{NSE}) = \int S(\omega)cos(\omega\tau_{NSE})d\omega \tag{10}$$

For a Lorentzian function, as has been used to model the spin-exciton in SmB_6,

$$S(\omega) = \frac{1}{\pi}\frac{\Gamma}{\Gamma^2 + \omega^2}, \tag{11}$$

and the integral gives $P(\tau_{NSE}) = \exp[-\Gamma\tau_{NSE}]$. By measuring the intensity of the scattering over several τ_{NSE}, the exponential can be fit to extract Γ. This exponential expression defines the envelope of the polarization and provides an estimate of the physical width Γ.

The spin echo data were collected at the R point, $\mathbf{Q}$ =[0.5 0.5 0.5], $k_f = 2.7$ Å^{-1}, and the crystal was aligned in the (-111) scattering plane. Typical count rates where 0.035/s, with signal/background=3/1. Scattering through spin excitations induces neutron spin flips; by utilizing parallel spin echo fields, only spin-flipped neutrons contribute to the spin echo signal. In the analysis, we assumed isotropic spin fluctuations and a non-spin-flip background. The line shape of $S(\omega)$ was assumed to be Lorentzian. To model the spin echo signals, a full simulation for the configuration used in the experiment was used, as described in Ref.[2,3].

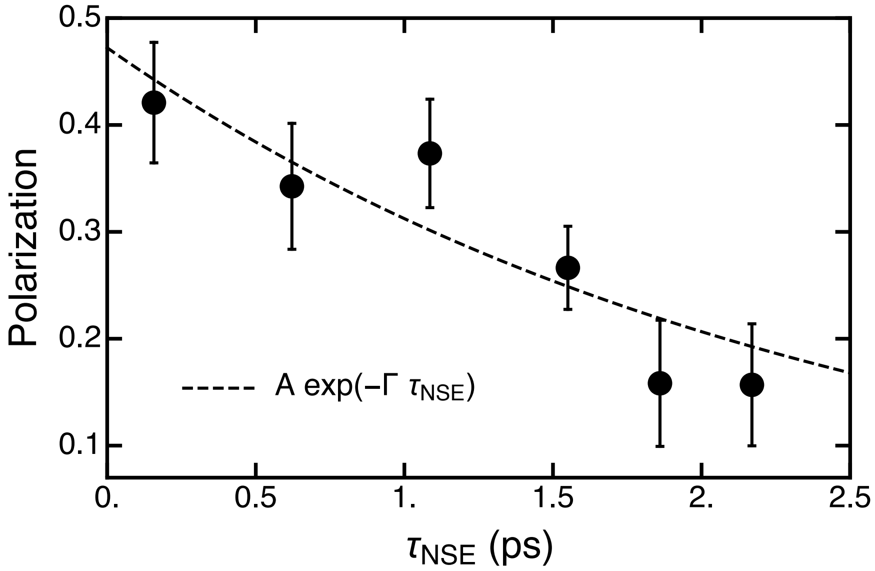

Supplementary Figure 5. Polarization of the 13 meV mode at 3.5 K as a function of neutron spin-echo time, τ_{NSE}.

Supplementary Note 3: Discussion

Wilson Ratio

In heavy Fermion metals, the DOS as well as the large effective mass conspire to produce a very large Sommerfeld constant (upwards of 1 J/mol-K^2). The dimensionless Wilson ratio, $R_W = \frac{4}{3}(\frac{\pi k_B}{g\mu_B})^2 \frac{\chi}{C/T}$, relates the susceptibility and specific heat. Systems with enhanced low temperature specific heat ($\gamma >> 1$ mJ/mol-K^2) and similarly enhanced susceptibility where $R_W \approx 1$ corresponds to a fermi liquid for which only the effective fermionic mass is renormalized. Isolated impurities cause little enhancement in the specific heat but contribute a Curie tail to the susceptibility, which would lead to a divergent Wilson ratio. The Wilson ratio in the 5% sample becomes approximately constant and of order 1 below 15 K. Given the substantial specific heat arising from 5% magnetic impurities, we take this as an indication of a Kondo-like effect for Gd impurities. The Wilson ratio becoming constant is evidence against isolated magnetic impurities.

Supplemental Note 4: Additional Equations

Model and thermodynamics

The "s-d" model given by the Hamiltonian

$$H = \int d^d k\, \varepsilon_{\mathbf{k}} \psi^\dagger_{\mathbf{k}} \psi_{\mathbf{k}} - J \sum_{i=1}^{N_i} \mathbf{S}_{\mathbf{r}} \cdot \psi^\dagger_{\mathbf{r}_i} \frac{\sigma}{2} \psi_{\mathbf{r}_i} \tag{12}$$

describes conduction electrons and localized magnetic moments in d dimensions coupled together by the Kondo term (J). The conduction electrons with energy dispersion $\varepsilon_{\mathbf{k}}$ are represented by creation and annihilation operators $\psi^\dagger_{\mathbf{k}}, \psi_{\mathbf{k}}$, while the local moments are captured by spin operators $\mathbf{S}_{\mathbf{r}}$ at randomly scattered positions $\mathbf{r}$. For simplicity, we treat local moments as spin $S = \frac{1}{2}$ degrees of freedom and assume

they are too far apart to mutually interact. We also assume a simple quadratic dispersion $\varepsilon_{\mathbf{k}} = k^2/2m$ for conduction electrons in calculations, but the final results depend only on their density of states $\eta(\mu)$ at the Fermi level μ and not on the precise dispersion, shape of the Fermi sea, number of bands or Fermi pockets, etc. Therefore, we anticipate that the results qualitatively capture the predominant features even if "conduction" electrons experience localization on large length-scales (e.g. Anderson localization). The partition function, Ξ, is the imaginary-time path-integral derived from the Hamiltonian (12) with Zeeman field $h = \gamma_{\text{eff}}\mu_{\text{B}}B$ applied in the $\hat{\mathbf{z}}$ direction. We take the conduction electrons and local moments to have the same magnetic moment. Thermodynamic quantities of interest are calculated from the relationships:

$$m = -\frac{\partial g}{\partial h} \quad , \quad s = -\frac{\partial g}{\partial T} \quad , \quad c = T\frac{\partial s}{\partial T} \, , \tag{13}$$

where

$$g = -\frac{kT}{V}\log(\Xi) + (-i\lambda)n_i + T\Delta s \, . \tag{14}$$

Here, V is the sample volume, $n_i = N_i/V$ the concentration of Kondo impurities, s entropy density and h the Zeeman energy. The partition function Ξ is calculated using the grand canonical ensemble, so we must explicitly add the $(-i\lambda)n_i$ term to treat the impurities canonically at constant number N_i ($-i\lambda$ will play the role of the impurity chemical potential). Our method generates additional unphysical constant entropy Δs, which is subtracted out.

For unperturbed free electrons and local moments, the contribution of decoupled local moments with concentration n_i is:

$$\begin{aligned} m_{\text{m}} &= n_i \tanh\left(\frac{\beta h}{2}\right) \\ c_{\text{m}} &= 2n_i k_{\text{B}} \left(\frac{\beta h}{2\cosh\left(\frac{\beta h}{2}\right)}\right)^2 \end{aligned} \tag{15}$$

at any temperature and magnetic field. The magnetization of local moments exhibits a linear dependence on small magnetic fields $\beta h \ll 1$ and saturates in large magnetic fields $\beta h \gg 1$ with the full unscreened moment. The isolated magnetic moments have no heat capacity at any temperature in the absence of magnetic field ($h = 0$), while an unconventional excess heat capacity dependent on n_i and not thermally activated is observed in the experiment. As such, our experimental result is inconsistent with trivial impurities and higher orders of perturbation are required to determine the relevant modifications to magnetization and zero-field heat capacity.

Perturbation theory

The perturbative expansion of the free energy in powers of $J\eta(\mu)$ is the sum of connected vacuum Feynman diagrams:

$$\log(\Xi) = \log(\Xi_{\text{c}}) + \log(\Xi_{\text{m}}) + \sum_{n=1}^{\infty} F_n \tag{16}$$

where Ξ_c and Ξ_m are the factorized grand canonical partition functions of the conduction electrons and local moments, respectively, and F_n is the nth order diagram. The bare propagators G of conduction electrons and D of local moments are given by matrices operating in the two-component spinor space:

$$\begin{aligned} G(\mathbf{k},\omega_n) &= \frac{1}{i\omega_n - (\varepsilon_\mathbf{k}-\mu) + h\sigma^z} \\ D^{ij}(\Omega_n) &= \frac{\delta_{ij}}{i\Omega_n - i\lambda + h\sigma^z}, \end{aligned} \tag{17}$$

where $i,j = 1,\dots,N_i$ enumerate impurity sites, and ω_n, Ω_n are fermionic Matsubara frequencies that take values $(2n+1)\pi \times k_\mathrm{B}T$ for integer n. The original Lagrange multipliers λ_i are replaced by their average purely imaginary value λ, so that $-i\lambda$ serves as the impurity electron chemical potential. The matrix elements of these propagators, indexed by $\alpha,\beta \in \pm 1$ spin-projection states along the $\hat{\mathbf{z}}$ axis are:

$$\begin{aligned} G_{\alpha\alpha'}(\mathbf{k},\omega_n) &= \frac{1}{2}\sum_{s=\pm1} \frac{\delta_{\alpha\alpha'} + s\sigma^z_{\alpha\alpha'}}{i\omega_n - (\varepsilon_\mathbf{k} - \mu - hs)} \\ D^{ij}_{\beta\beta'}(\Omega_n) &= \frac{\delta_{ij}}{2}\sum_{s=\pm1} \frac{\delta_{\beta\beta'} + s\sigma^z_{\beta\beta'}}{i\Omega_n - i\lambda + hs}\ . \end{aligned} \tag{18}$$

The bare vertex function for the Kondo coupling at $\omega + \Omega = \omega' + \Omega'$ is:

$$\begin{aligned} V_{\alpha\alpha'\beta\beta'}(\omega,\mathbf{k};\omega',\mathbf{k}';i,\Omega;j,\Omega') &= \frac{J}{2\beta}\delta_{ij}e^{i(\mathbf{k}-\mathbf{k}')\mathbf{r}_i}\sigma_{\alpha\alpha'}\sigma_{\beta\beta'} \\ &= \frac{J}{2\beta}\delta_{ij}e^{i(\mathbf{k}-\mathbf{k}')\mathbf{r}_i}(2\delta_{\alpha\beta'}\delta_{\beta\alpha'} - \delta_{\alpha\alpha'}\delta_{\beta\beta'})\ . \end{aligned} \tag{19}$$

Ultimately, after obtaining the partition functions, we use the standard thermodynamic relationships (13) to find the magnetization and specific heat to first order:

$$\begin{aligned} m &= m_\mathrm{c} + n_i\left[(1+4\alpha)\tanh\left(\frac{\beta h}{2}\right) + \frac{2\alpha\beta h}{\cosh^2\left(\frac{\beta h}{2}\right)}\right] \\ c &= c_\mathrm{c} + \frac{n_i k_\mathrm{B}(\beta h)^2}{2\cosh^2\left(\frac{\beta h}{2}\right)}\left[1 + 8\alpha - 4\alpha\beta h \tanh\left(\frac{\beta h}{2}\right)\right] \end{aligned} \tag{20}$$

up to the linear order in

$$\alpha = \frac{J\eta(\mu)}{8}\left[1 + \frac{(d-2)(d-4)}{8}\frac{(\beta h)^2 + \pi^2}{3(\beta\mu)^2} + \cdots\right]\ ,$$

where m_c and c_c are the free Fermi gas contributions and d is the dimensionality. Additional temperature and field dependence enters through α, but in general the series in parenthesis is ~ 1, and exactly 1 in $d = 2$ dimensions.

An inspection of the formula (20) for magnetization reveals that an antiferromagnetic Kondo coupling $\alpha < 0$ effectively reduces the impurity magnetic moment, suppresses magnetization at low fields, and pushes the magnetization saturation to higher fields – all due to the formation of Kondo singlets that resist the externally applied field. Some of these effects can be weak, but deviations from the free moment

magnetization (15) seen in the experiment are largely consistent with (20). If we take α to be constant, then the impurity magnetization in (20) is controlled by a single scale βh, which is inconsistent with experimental results. More realistically, the effective Kondo coupling $|\alpha|$ gradually increases in $d=3$ when h/μ or $k_{\mathrm{B}}T/\mu$ are small, indicating an enhanced ability of conduction electrons to screen the spin of local moments. The importance of scales other than βh become more visible at higher orders of perturbation theory. Still, the behavior of these expressions at low temperatures is unphysical due to the breakdown of perturbation theory. The qualitative feature of the second order result as related to the main text is the featureless rise in specific heat towards lower temperatures. This rise is absent for paramagnetic impurities, but a distinctive feature in the specific heat of the s-d Kondo model.[4,5]

References

1. Tan, B. *et al.* Unconventional Fermi surface in an insulating state. *Sci.* **349**, 287–290 (2015).

2. Groitl, F., Keller, T., Rolfs, K., Tennant, D. A. & Habicht, K. Anomalous thermal decoherence in a quantum magnet measured with neutron spin echo spectroscopy. *Phys. Rev. B* **93**, 134404 (2016).

3. Tseng, K., Keller, T., Walters, A., Birgeneau, R. & Keimer, B. Neutron spin-echo study of the critical dynamics of spin-5 2 antiferromagnets in two and three dimensions. *Phys. Rev. B* **94**, 014424 (2016).

4. Yosida, K. *Theory of Magnetism*, vol. 122 (Springer Science & Business Media, 1996).

5. Tari, A. *The specific heat of matter at low temperatures* (World Scientific, 2003).
